# Protective Effect of Quercetin on Oxidative Stress in Glucose-6-Phosphate Dehydrogenase-Deficient Erythrocytes *in Vitro*

**Published:** 2010

**Authors:** Akram Jamshidzadeh, Abbas Rezaeian Mehrabadi

**Affiliations:** *Pharmaceutical Research Center, Shiraz University of Medical Sciences, Shiraz, Iran.*

**Keywords:** Quercetin, G6PD-deficiency, Oxidative stress, Oxygen-free radical scavenger, Erythrocytes, Vitamin C

## Abstract

Glucose-6-phosphate dehydrogenase (G6PD) deficient subjects are vulnerable to oxidative stress. Quercetin, a flavonoids, has been employed as a potent oxygen-free radical scavenger in order to assess the protective effects of quercetin against H_2_O_2_-induced oxidative damage in G6PD-deficient and normal human erythrocytes.

Erythrocytes of G6PD-deficient (n = 10) and normal (n = 10) subjects were incubated with different concentrations of quercetin. The produced thiobarbituric acid reactive substance (TBARS) and glutathione (GSH) level of erythrocytes were then subsequently measured.

Different concentrations of quercetin showed no significant hemolysis, compared with the phosphate buffer solution. Upon challenge with H_2_O_2_, there was a significant (p < 0.005) decrease in GSH and an increase in TBARS level in G6PD-deficient erythrocytes. With quercetin, it managed to preserve concentrations of 15 to 75 mM preserved GSH and TBARS levels of normal and G6PD-deficient erythrocytes against H_2_O_2_-induced oxidative damage.

In addition to its well-established antioxidant effects, quercetin was also found to have cytoprotective properties.

## Introduction

Glucose-6-phosphate-dehydrogenase deficiency is a hereditary disorder with higher potential for oxidative damage, due to chronic redox imbalance in red blood cells, that often results in clinical manifestation of mild to severe haemolysis in patients with these disorders. G6PD deficient subjects are at high risk of developing haemolytic anemia, when exposed to exogenous oxidative agents (1). G6PD deficiency-related haemolysis is particularly serious in the neonates, as the excessive unconjugated bilirubin released from haemolysed erythrocytes can pass through the blood brain barrier and enter the brain, thereby causing neurological complications including mental retardation, convulsion, cerebral palsy, hearing deficit, or even death (2) .

The most effective management strategy for G6PD deficiency is to prevent haemolysis, by avoiding oxidative stressors (such as drugs and *Vicia faba *beans). This approach, however, requires the patient to be aware of their deficiency, as a result of a previous haemolytic episode or a screening program. Fortunately, acute haemolysis in G6PD-deficient individuals is usually short lived, and does not need a specific treatment. In rare cases (usually children), acute haemolysis leading to severe anemia would require transfusion of red blood cells (3). 

In organisms, hydrogen peroxide (H_2_O_2_) is naturally produced as a by- product of oxygen metabolism; hence, virtually all possess enzymes known as peroxidases. H_2_O_2_ is useful for inducing experimental oxidative stress in the cells and for studying its oxidative status in physiological and pathological situations. Several studies have described the antioxidant activities of flavonoids, as hydrogen-donors and free-radical scavengers. These observations raise the possibility of using flavonoids as therapeutic drugs to prevent oxidative alterations in G6PD-deficient erythrocytes. Antioxidant agents such as selenium, vitamin C and α-tocopherol have been shown to prevent the haemolytic crises of G6PD deficient subjects (4-7). *Ginkgo biloba *extract has been reported to have antioxidant activity and to prevent oxidative damage in erythrocytes. Its antioxidative capacity has been reported to be more effective than vitamin C (8, 9). Quercetin (3, 30, 40, 5, 7-pentahydroxylflavone) is a typical flavonoid, present in fruits and vegetables, and has attracted a great deal of attention as a potential antioxidant (10). There has been no report of known adverse effects from the use of quercetin in the published medical literature (11, 12). However, its antioxidative effect has not been studied in G6PD deficiency.

The aim of this study was to investigate the protective effects of quercetin against H_2_O_2_-induced oxidative damage in human erythrocytes. Antioxidant status was examined and the potential application of quercetin, compared and contrasted with vitamin C.

## Experimental

This study was approved by the Clinical Research Ethics Committee, Shiraz University of Medical Sciences. All chemicals were purchased form Merck Chemical Company, with the best and purest grade available.

*Subject recruitment*

Human peripheral blood samples (20 mL) collected by venipuncture from 10 (mean age of 19 ± 8 years) G6PD-deficient and 10 healthy matched donors, with informed consents, were stored in adenine citrate dextrose at 4 °C. All analyses were carried out within 24 h. 

*G6PD screening*

Qualitative screening of G6PD was carried out based on the Fairbanks and Klee method (13). Blood samples obtained were centrifuged at 3000 rpm for 10 min at 22 °C, then they were washed three times with 0.9% w/v saline and centrifuged ( at 3000 rpm for 10 min) to obtain samples of constantly packed cells. Finally, erythrocyte suspensions (10% v/v) were prepared in pH 7.4 phosphate buffered saline (PBS).

Ten microliters of different blood samples were individually added to tubes containing 100 μl of a solution consisting of 50 mM glucose-6-phosphate, 7.5 mM NADP^+^, 0.5 g/dL saponin, 4 mM oxidized glutathione and 0.7 M of pH7.8 tris–HCl buffer. The blood samples were individually mixed and then 10 μL aliquots immediately transferred onto a 1×1 cm filter paper. After 5 min, this procedure was repeated. Spots were allowed to dry and then examined under a long-wave (320–400 nm) 150 W lamp. Florescence of specimens was compared with positive and negative controls.

The presence of Heinz bodies in freshly drawn blood samples were observed in order to approve G6PD deficient red blood cells after challenging with 0.1mg/mL α–naphthol. 

Hb level and RBC count were also measured, using a DAX-48 autoanalyzer in all subjects.

*Incubation of RBC suspension with H*_2_ *O*_2_ *and quercetin*

2.5 mL of the normal and G6PD-deficient erythrocyte suspension samples were treated with quercetin (0.1-150 mM) and vitamin C (250 mM) for 2h (1h before and 1h after incubation with 20 mM of H_2_O_2_) at 37 °C. After 1 h of H_2_O_2_ treatment, levels of TBA-reactive substances (TBARS) and GSH content were measured.

*Determination of lipid peroxidation*

The lipid peroxidation of red blood cell (RBC) membrane was assessed, as described by Stocks and Dormandy (14). After a 2 h incubation period of erythrocytes with quercetin and vitamin C in the absence or presence of H_2_O_2_ (20 mM) at 37 °C, 0.5 mL of 25% TCA was added to 1 mL of suspension and the mixture was centrifuged at 1000 g for 5 min. 

To 1 mL of the resulting supernatant, 1 mL of 1% thiobarbituric acid (TBA) in 0.05 M NaOH was added followed by boiling for 15 min. The formation of TBARS was used as a measure of lipid peroxidation. The TBARS concentration was determined using a spectrophotometer (BioTek Instruments, Winooski, VT, USA) at 532 nm and the results were expressed as ng MDA/g of haemoglobin.

*Determination of GSH*

Reduced glutation (GSH) was determined, based on the method of Ellman (15). A 1 mL aliquot of the erythrocytes (0.5 mL of cells percipited by 2 mL of 5% TCA) was taken and 0.5 mL of Ellman’s reagent (0.0198% DTNB in 1% sodium citrate) and 3mL of phosphate buffer (pH 8.0) were added. The developed color was then read at 412 nm. The GSH concentrations in test samples were calculated, with reference to the standard curve of GSH (1–100 μg/mL). The results were expressed as μg/g of haemoglobin.

*Statistical analysis*

All the experiments were carried out at least in triplicate, using RBC from 10 subjects. Results were tested by one-way ANOVA, followed by usig the Dunnett post-hoc test. The difference between the G6PD-deficient and normal groups was analyzed by student’s t-test. All the statistical tests were carried out at a 5% level of significance (p < 0.05). Data obtained have been expressed as mean ± SD.

## Results

The mean RBC count was 5.2 ± 0.03 million/μL in the control and 4.6 ± 0.07 million/μL in the G6PD deficient group. The mean haemoglobin content was 13.93 ± 0.2 g/dL in the control group and 11.3 ± 0.8 g/dL in the deficient group, respectively (data not shown). GSH content and TBARS values were not found to be significant in G6PD deficient and normal erythrocytes. Upon challenge with H_2_O_2_ (1-100 mM), there was a significant decrease in GSH levels (p < 0.005) and an increase in TBARS levels in G6PD deficient and normal erythrocytes (Table 1). 

**Table 1:** Cytotoxicity of H_2_O_2_ in normal and G6PD-deficient erythrocytes. 2.5 mL volumes of the normal and G6PD-deficient erythrocyte suspension samples were treated with different concentrations of H_2_O_2_ for 1h at 37 °C. After 1 h, TBARS levels and GSH content were measured as described in the experimental section. Results are mean ±SD of 10 different subjects

**Addition**	**Normal**		**G6PD-deficient **	
	**ng MDA/g Hb **	**μg GSH/g Hb**	**ng MDA/g Hb**	**μg GSH/g Hb**
Control	33±2	275±25	37±6	340±44
H_2_O_2_ (0.1 mM)	37±4	271±15	40±2	345±21
H_2_O_2_ (0.5 mM)	35±8	263±32	44±5	331±35
H_2_O_2_ (1 mM)	51±2*	210±28*	62±6*	267±33*
H_2_O_2_ (20 mM)	125±8**	145±28*	177±6**	200±23*
H_2_O_2_ (50 mM)	138±42**	116±34**	189±12**	145±26**
H_2_O_2_ (100 mM)	132±28**	108±42**	123±31**	141±35**

*: Significantly different from the control group (p < 0.05).

**: Significantly different from the control group (p < 0.01).

**Figure 1:**
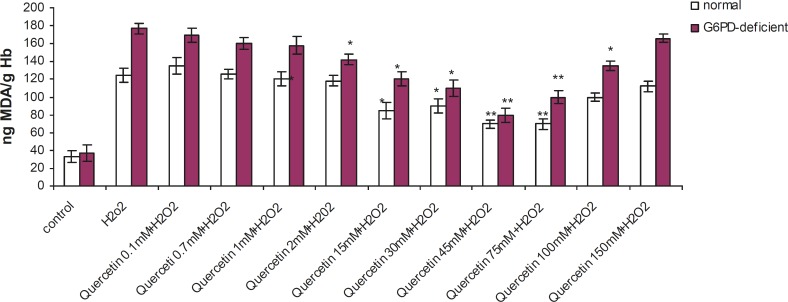
Effect of quercetin on lipid peroxidation induced by H_2_O_2_ in normal and G6PD-deficient erythrocytes. 2.5 mL volumes of the normal and G6PD-deficient erythrocyte suspension samples were treated with quercetin (75 mM) for 2h (1h before and 1h after incubation with 20 mM of H_2_O_2_) at 37 °C. After 1 h of H_2_O_2_ treatment, TBARS levels were measured as described in the experimental section. Results are mean + SD of 10 different subjects. *: Significantly different from the H_2_O_2_–treated group (p < 0.05). **: Significantly different from the H_2_O_2_–treated group (p < 0.01).

**Figure 2:**
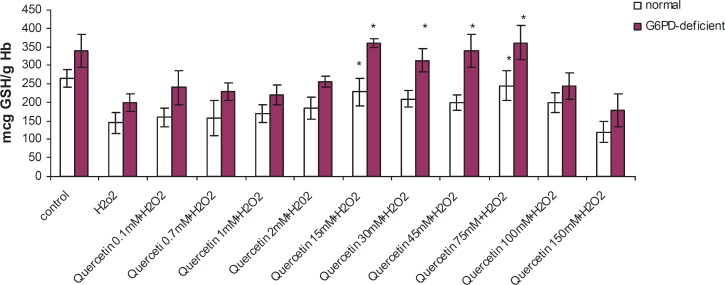
Effect of quercetin on glutathione depletion induced by H_2_O_2_ in normal and G6PD-deficient erythrocytes. 2.5 mL volumes of the normal and G6PD-deficient erythrocyte suspension samples were treated with quercetin (75 mM) for 2h (1h before and 1h after incubation with 20 mM of H_2_O_2_) at 37 °C. After 1 h of H_2_O_2_ treatment, GSH content was measured as described in the experimental section. Results are mean ± SD of 10 different subjects. *: Significantly different from the H_2_O_2_–treated group (p < 0.05). **: Significantly different from the H_2_O_2_–treated group (p < 0.01).

Incubation of RBC with quercetin (15-75 mM) for 2 h caused a significant decrease in TBARS levels and an elevation in GSH levels (p < 0.005) in G6PD deficient and normal erythrocytes. The protective effect of quercetin in the G6PD deficient group was higher than that of the normal subjects (Figures 1 and 2). As shown in these figures, the protective effect of quercetin was dose-dependent and concentrations higher than 75 mM did not show any protective effect against H_2_O_2_. Furthermore, quercetin itself caused toxicity towards RBC to concentrations of 100 mM and higher (data not shown).

Quercetin was also more efficient than vitamin C in protecting against lipid peroxidation and GSH depletion in G6PD deficient and normal erythrocytes (Table 2). 

**Table 2:** Protective effect of quercetin and vitamin C against H_2_O_2_-induced cytotoxicity in normal and G6PD-deficient erythrocytes. 2.5 mL volumes of the normal and G6PD-deficient erythrocyte suspension samples were treated with quercetin (75 mM) and vitamin C (250mM) for 2h (1h before and 1h after incubation with 20 mM of H_2_O_2_) at 37 °C. After 1 h of H_2_O_2 _treatment, TBARS levels and GSH content were measured as described in the experimental section. Results are mean + SD of 10 different subjects

**Addition **	**Normal **		**G6PD-deficient **	
	**ng MDA/g Hb **	**μg GSH/g Hb **	**ng MDA/g Hb **	**μg GSH/g Hb **
Control	33±2	275±25	37±6	340±44
H_2_O_2_ (20 mM)	125±8	145±28	177±6	200±23
H_2_O_2_+ vitamin C (250 mM)	65±8*	110±32*	123±41*	310±26*
H_2_O_2_+ quercetine (75 mM)	70±6 **	245±40**	100±7*	362±45**

*: Significantly different from H_2_O_2_–treated group (p < 0.05).

**: Significantly different from H_2_O_2_–treated group (p < 0.01).

## Discussion

There is considerable current interest in the cytoprotective effects of natural antioxidants against oxidative stress and the different defense mechanisms involved (16). This study demonstrates that quercetin, a common dietary flavonoid with a high in-vitro antioxidant activity, has the ability to protect the erythrocytes against an oxidative insult by modulating reduced glutathione concentration and MDA production in the cell. 

Enhanced susceptibility against oxidative stress of G6PD-deficient erythrocytes has been reported by several authors (17-19). Various studies have indicated that vitamin E, with and without selenium, can significantly improve the haematological manifestation of the patients with G6PD deficiency (4, 7). Our findings have shown that prior and concomitant administration of vitamin C and quercetin, along with an oxidant agent, can significantly reduce the peroxidative damage of erythrocytes in G6PD-deficient subjects. The antiperoxidative effect of quercetin (depicted in Figure 1) showed that 75 mM of quercetin inhibited the formation of TBARS by 56% (from 177 to 100 ng MDA/gHb in G6PD deficient samples). Prior and concomitant addition of quercetin, along with H_2_O_2_, increased GSH levels to 245 and 362 μg GSH/g Hb in normal and deficient erythrocytes, respectively. The antiperoxidative effect of quercetin was also found to be greater than vitamin C. Our findings also indicated that despite its preventive effect in peroxidative conditions, quercetin showed an oxidant characteristic when it was applied directly to erythrocytes in high doses (100 mM and higher). These results are in accordance with the reports on the pro-oxidative activities of some flavonoids on various cancer cell lines (20-22). The pro-oxidative effect of 18 commonly used Chinese herbal remedies on human G6PD-deficient red blood cells has been evaluated by Ko *et al*. They presented the first evidence on the pro-oxidative action of six different Chinese herbal remedies (Rhizoma Coptidis, Cortex Moutan, Radix Rehmanniae, Rhizoma Polygoni Cuspidati, Radix Bupleuri and Flos Chimonanthi) on G6PD-deficient blood samples (23).

Quercetin is converted to *o*-quinone by autocatalytic oxidation and the *o*-semiquinone radical is inevitably produced as a reactive intermediate (24). Oxygen molecules can react with this radical, resulting in the production of O_2_ and H_2_O_2_. These reactive oxygen species (ROS) affect cellular redox signaling pathways and are capable of inducing cellular oxidative damage (25). It is therefore possible that quercetin can act as both a pro-oxidant and an anti-oxidant. This paradoxical phenomenon may explain the reason why quercetin accelerates cellular oxidative damage and/or acts as a mutagen, instead of an anti-mutagen, in a variety of in-vitro studies (26).

Data presented here show that quercetin has protective effects on GSH levels and TBARS. Reduced glutathione (GSH) is the main non-enzymatic antioxidant defense within the cell, reducing different peroxides, hydroperoxides and radicals (alkyl, alkoxyl, peroxyl, etc.) (27). It is usually assumed that GSH depletion reflects intracellular oxidation. On the contrary, an increase in GSH concentration could be expected to prepare the cell against a potential oxidative insult (28-30). Furthermore, it has been reported that GSH levels were significantly lower in G6PD-deficient samples, when compared with the G6PD-normal RBC (31). Based on these findings, it can be concluded that cytoprotective activity of quercetin is related to the increase in the reduced glutathione of G6PD-deficient erythrocytes. Considering the high incidence of G6PD deficiency in specific geographic locations such as Southeast Asia, Mediterranean region and parts of Africa, our findings can be foreseen to *in-vivo *studies aimed at design of suitable flavonoid-based drugs of importance in preventing free radical induced peroxidation and lysis of erythrocytes.
